# Atomistic Study of Mechanical Behaviors of Carbon Honeycombs

**DOI:** 10.3390/nano9010109

**Published:** 2019-01-18

**Authors:** Huaipeng Wang, Qiang Cao, Qing Peng, Sheng Liu

**Affiliations:** 1 The Institute of Technological Sciences, Wuhan University, Wuhan 430072, China; wanghuaipeng@whu.edu.cn; 2 Nuclear Engineering and Radiological Sciences, University of Michigan, Ann Arbor, MI 48109, USA; 3 School of Power and Mechanical Engineering, Wuhan University, Wuhan 430072, China; victor_liu63@126.com

**Keywords:** carbon honeycomb, molecular dynamics, LAMMPS, uniaxial tension, nanoindentation

## Abstract

With an ultralarge surface-to-volume ratio, a recently synthesized three-dimensional graphene structure, namely, carbon honeycomb, promises important engineering applications. Herein, we have investigated, via molecular dynamics simulations, its mechanical properties, which are inevitable for its integrity and desirable for any feasible implementations. The uniaxial tension and nanoindentation behaviors are numerically examined. Stress–strain curves manifest a transformation of covalent bonds of hinge atoms when they are stretched in the channel direction. The load–displacement curve in nanoindentation simulation implies the hardness and Young’s modulus to be 50.9 GPa and 461±9 GPa, respectively. Our results might be useful for material and device design for carbon honeycomb-based systems.

## 1. Introduction

Graphene is known widely, due to its excellent mechanical nature, as a so-called “miracle material”, with many of its characteristics measured experimentally or theoretically exceeding those obtained in other materials: A Young’s modulus of 1 TPa and intrinsic strength of 130 GPa [1,2], high stiffness [3] and fracture strain [4,5], a normal-auxeticity mechanical phase transition [5], etc. Due to these predominant properties, graphene has promising potential to be employed in various applications: Paints and coatings of nanocomposites [4,6], flexible electronics [7], and bioapplications [8,9,10]. Nevertheless, considering the difficulty in engineering synthesis of large-area and high-quality graphene, so far, there is little practical application of industrially produced graphene. Consequently, it is worth focusing on allotropes of graphene [11,12,13], which are more stable and feasible to produce. Recently, a stable carbon allotrope, namely, carbon honeycomb (CHC), a three-dimensional graphene, was synthesized. There are a few reports on of its physical absorption [14,15], electronic band structure [16], thermoelectric performance [17], thermal transport properties [18,19], and phonon properties [15]. 

According to the cell patterns in the plane perpendicular to the cell axis, carbon honeycombs can be categorized into two sets: Armchair CHC (ac_n_ CHC) and zigzag CHC (zz_m_ CHC) [20]. When the number of armchair or zigzag lines (n in ac_n_ or m in zz_m_) varies, the cell sizes of CHCs and, thus, their mechanical properties change correspondently. Krainyukova et al. [14] synthesized this carbon allotrope by deposition of vacuum-sublimated graphite and proposed periodic and random structures of CHCs. Regarding the mechanical properties of CHC, some analytical studies have been carried out [16,19,20,21,22,23,24]. Karfunkel et al. first proposed a family of hypothetical zz_m_–sp^2^–sp^3^ CHC structures and revealed that the carbon modifications are as stable as diamond by solid-state semi-empirical SCF methods using the modified neglect of diatomic overlap (MNDO) Hamiltonian [23]. Park et al. studied the mechanical properties of zz_m_–sp^2^–sp^3^ CHCs with different m (where m can be an integer or a half integer) using ab initio pseudopotential as well as the environment-dependent tight-binding method, making clear that the carbon allotrope is elastically stable and has a fairly high shear modulus [24]. Pang et al. made a systematical analysis of failure strength and strong anisotropic Poisson’s effect of ac_n_–sp^2^–sp^3^ CHCs with different cell sizes via molecular dynamics simulation using optimized reactive empirical bond-order potential [19]. Gu et al. used molecular dynamics (MD) simulation with modified reactive empirical bond-order potential to give the stress–strain curves of symmetrical and asymmetrical ac_2_–sp^2^–sp^3^ CHCs, ac_3_–sp^2^–sp^3^ CHCs, and zz_2_–sp^2^–sp^3^ and zz_2.5_–sp^2^–sp^3^ CHCs [21]. Zhang et al. [16] built the in-plane compression and out-of-plane nanoindentation tests of ac_n_–sp^2^ and zz_m_–sp^2^–sp^3^ CHCs via MD analysis with the Adaptive Intermolecular Reactive Empirical Bond Order potential (AIREBO) [25] potential. Meng et al. investigated the out-of-plane compression behaviors of both ac_n_–sp^2^–sp^3^ and zz_m_–sp^2^–sp^3^ CHCs using MD simulation with AIREBO potential [20]. Despite these studies, a full and clear understanding of the inner mechanism of the deformation of CHCs is still lacking but desirable due to its various promising applications as an engineering material. In this study, we used MD simulation with AIREBO potential to investigate the transformation from an ac_2_–sp^2^–sp^3^ CHC to an ac_2_–sp^2^ CHC, which causes the change of tensile deformation behavior of ac_2_ CHCs. In the meanwhile, we gave the stress–strain curves, Young’s modulus, and Poisson’s ratio of ac_2_ CHCs, which show a great agreement with previous studies. We also built an out-of-plane nanoindentation test to investigate the plastic deformation behaviors of ac_2_–sp^2^–sp^3^ CHCs, giving the hardness and Young’s modulus. 

## 2. Crystal Structure of Carbon Honeycomb

**Figure 1:**
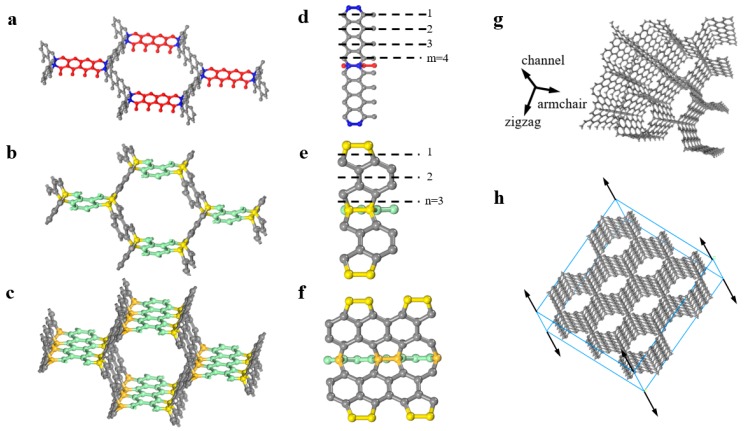
Honeycomb structures in (**a**) zigzag sp^2^–sp^3^-bonded carbon honeycomb (zz_4_–sp^2^–sp^3^ CHC), (**b**) symmetrical armchair sp^2^–sp^3^-bonded carbon honeycomb (sym-ac_3_–sp^2^–sp^3^ CHC), and (**c**) asymmetrical armchair sp^2^–sp^3^-bonded carbon honeycomb (asym-ac_3_–sp^2^–sp^3^ CHC). (**d**), (**e**), and (**f**) are the zoom-in views exhibiting detailed carbon patterns and bonding ways of hinge atoms shown in (**a**), (**b**), and (**c**), respectively. For convenience, a coordinate system attached to sym-ac_3_–sp^2^–sp^3^ CHC is displayed in (**g**). Uniaxial tension is applied on sym-ac_3_–sp^2^ and sym-ac_3_–sp^2^–sp^3^ CHCs along the axis of cell channel. All the simulation supercells are cubic and periodical in three directions.

Carbon honeycomb, just as its name implies, is a kind of tubular structure with a honeycomb-like pattern through a top–down perspective. The tube wall can be regarded as a graphene-like monolayer, thereby deemed to be a 3D version of graphene. One thing about its structure that remains to be discussed is whether three adjacent tube walls are bound up with each other via sp^3^ or sp^2^ bonding (Figure 1b,c). Undoubtedly, atoms that build up the tube shells are combined with neighboring carbon atoms by an sp^2^ bond, as is the case in graphene, whereas those who pull contiguous graphene-like walls together, which are called hinge atoms, give rise to more options, one kind of typical sp^3^ bonds with non-hinge atoms (tube atoms) or one variant of diamond-like sp^3^ bonds with both adjacent hinge and non-hinge atoms. In the case of sp^2^ bonds, hinge atoms do not interact with each other, allowing for electron exchange with only adjacent tube atoms. In the case of sp^3^ bonds, it is not only nearby tube atoms but also nearest-neighbor hinge atoms that get bonded with the center hinge atom, resulting in a diamond-like atom-hinge that ties up three around the atomic walls. According to the carbon patterns in the plane perpendicular to the axis of cell channel, carbon honeycombs can be categorized into two sets: Armchair CHC (ac_n_ CHC) and zigzag CHC (zz_m_ CHC) [20], as shown in Figure 1. Furthermore, carbon honeycombs can be subdivided into sp^2^ and sp^2^–sp^3^ CHCs. In zz_m_ CHCs, carbon atoms of graphene-like walls are bonded by sp^2^ bonds, while hinge atoms can be bonded with adjacent atoms only by sp^3^ bonds. Therefore, zz_m_ CHCs always refer to zz_m_–sp^2^–sp^3^ CHCs. However, the other case is different. In ac_n_ CHCs, hinge atoms can be bonded with atoms around them by sp^2^ or sp^3^ bonds. Therefore, ac_n_ CHCs can be subdivided into ac_n_–sp^2^ CHCs and ac_n_–sp^2^–sp^3^ CHCs. In the case of ac_n_–sp^2^–sp^3^ CHCs, hinge atoms can be distributed symmetrically or asymmetrically in a crystal cell, according to which ac_n_–sp^2^–sp^3^ CHCs can also be subdivided into a symmetrical one and asymmetrical one, as shown as Figure 1b,c,e,f. 

**Figure 2:**
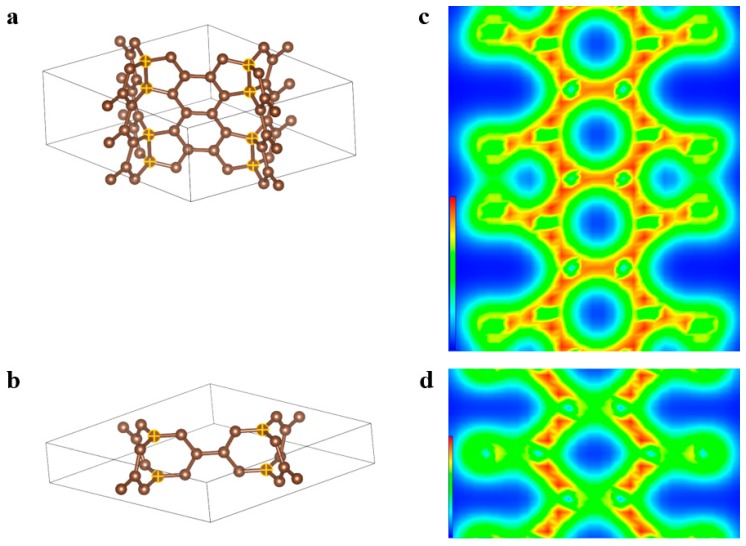
The primitive cells of sym-ac_3_–sp^2^ and sym-ac_3_–sp^2^–sp^3^ CHCs are shown in (**a**) and (**b**), respectively. (**c**) and (**d**) display the slice contours of electron density from density functional theory (DFT) calculation of structures shown in (**a**) and (**b**), respectively.

Herein, we implemented a density functional theory (DFT) calculation for both primitive cells of sym-ac_3_–sp^2^ and sym-ac_3_–sp^2^–sp^3^ CHCs, as shown in Figure 2b,c, using PWmat [26,27], to examine their stabilities. For both types, cell relaxation and atom relaxation were exerted in subsequence to determine their respective energy-minimized configurations. Subsequently, self-consistent calculation was carried out to optimize the electron interaction in both primitive cells, as visualized in Figure 1d,e. As for the sym-ac_3_–sp^2^–sp^3^ CHC, stronger sp3 bonds between the hinge atom and nearest-neighbor tube atoms can be observed than those between two adjacent hinge atoms, the bond angle of which is measured as 103.35°. Meanwhile, exactly strong sp^2^ bonds among graphene-like atoms can be distinguished from sp^3^ bonds, with denser electron density around tube atoms observed (red-yellow hexagons in Figure 1d). As for the sym-ac_3_–sp^2^ CHC, hinge atoms are not attached to each other, with weaker sp2 bonds between hinge and non-hinge atoms observed than those between only tube atoms. The DFT calculation results, i.e., that the cohesive energy is –154.19 eV/atom of the sym-ac_3_–sp^2^ CHC, –154.84 eV/atom of sym-ac_3_–sp^2^–sp^3^ CHC, respectively, indicate that two types of configurations are both likely to exist stably. Additionally, the slightly stronger cohesive interaction implies the sym-ac_3_–sp^2^–sp^3^ CHC structure is relatively more stable. 

## 3. Molecular Dynamics Simulations

We used molecular dynamics simulations to do the investigations of mechanical behaviors of the sym-ac_3_–sp^2^ CHC and sym-ac_3_–sp^2^–sp^3^ CHC. The reason we focused on them is that in the work of Krainyukova et al. [14], a periodic carbon structure identical to the sym-ac_3_–sp^2^ CHC according to their synthetic CHC samples, proving that ac_3_ CHCs are more likely to exist in the nature. The method of MD simulations is well established to investigate the elastic and plastic behaviors of sym-ac_3_–sp^2^ and sym-ac_3_–sp^2^–sp^3^ CHCs. Initially, we carried out uniaxial tension tests for both types of CHCs, simultaneously determining the Young’s moduli of the sym-ac_3_–sp^2^–sp^3^ CHC to be 551 ± 4 GPa and of sym-ac_3_–sp^2^ CHC to be 542±4 GPa in the channel direction (Figure 1g,h), following which we carried out tension tests in the channel direction of two kinds of CHCs, giving stress–strain curves, respectively. Interestingly, a transformation of hinge atoms from sp^3^ to sp^2^ was observed on the atomic scale, which can account for the yield stage that can be observed in the sym-ac_3_–sp^2^–sp^3^ CHC, while cannot be observed in the sym-ac_3_–sp^2^ CHC. Furthermore, a nanoindentation simulation was devised and the load–displacement curve was plotted to determine the hardness and Young’s modulus of the CHC, which are 50.9 GPa of hardness and 461 ± 9 GPa of Young’s modulus, respectively. Meanwhile, we discussed the plastic behavior of CHC in the process of nanoindentation. 

We used a molecular dynamics (MD) software, LAMMPS [28], which is an open-source code, to simulate two typical mechanical tests, uniaxial tension tests of the sym-ac_3_–sp^2^ CHC and sym-ac_3_–sp^2^–sp^3^ CHC and nanoindentation of the ac_3_–sp^2^–sp^3^ CHC. As for the tension simulation, given that we have set the boundary conditions as periodic in three directions, and thus, the mechanical response of the system is independent from the simulation scale, in order to accelerate the calculation, the simulation box was chosen as 3.408 nm × 2.951 nm × 4.181 nm, containing 2856 atoms. Both end-faces perpendicular to the direction of tension were freed from all kinds of forces. The whole system was minimized for energy and relaxed at specified temperatures (10 K and 300 K) and zero pressure in the NPT ensemble (where the number of particles, pressure, and temperature are constant) using AIREBO [25] potential (cutoff radius = 2.5 Å), to eliminate inner stress. The strain rate is set as 0.01 ps^−1^. Considering that temperature effect will cause random distribution of atoms, which will prevent us from observing the bonding transformation of hinge atoms, in addition to at room temperature, uniaxial tension tests were also carried out at 10K, at which little thermal motion is allowed so that a perfect configuration might remain. 

We also simulated the entire process of nanoindentation on an ac_3_–sp^2^–sp^3^ CHC along the channel direction. A thick CHC plate measured as 17.82 nm × 15.43 nm × 11.60 nm was set up in a simulation box with a vacuum layer 9.00 nm thick in addition (Figure 2). The indenter is a cubic diamond sphere, 8.00 nm in diameter, with [001] direction aligned with the channel direction. The velocity of this indenter is set at 0.25 Å/ps. There is a 0.50-nm-thick layer on the bottom of the slab of CHC, all forces on which are zeroed. Nonperiodic conditions were selected in three directions in order to simulate an isolated system. Before the mechanical test, an energy minimization had been finished and the total ensemble had been relaxed efficiently without applied stress at 300 K, using an NVT ensemble (where the number of particles, volume and temperature are constant) and NPT ensemble with zero pressure successively, with AIREBO-morse [29] potential (cutoff radius = 2.5 Å). 

## 4. Results and Discussion

### 4.1. Uniaxial Tension

#### Strain–Stress Curve

**Figure 3:**
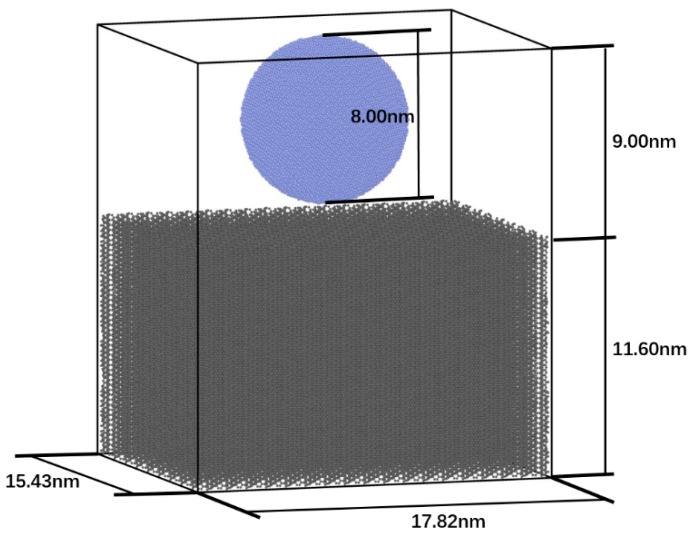
Schematic of simulation model of nanoindentation along the channel direction.

From uniaxial tension, we obtained the engineering strain–stress curves of both the sym-ac_3_–sp^2^ CHC and sym-ac_3_–sp^2^–sp^3^ CHC (Figure 3), which can depict the elastic pattern directly and characterize the plastic properties indirectly, gaining an insight into the similarities and differences between the mechanical behaviors of two kinds of CHCs. Our results are in great agreement with the stress–strain curves from a previous study [21], in which the fracture strength and strain equal to our results are exhibited. Although the stress–strain curves of ac_n_–sp^2^ CHCs with different sizes have been studied systematically, there have been a limited number of studies of the yield process, i.e., the transformation from sp^3^ to sp^2^, which is what we focus on. We also calculated the Young’s moduli of both CHCs, 542 ± 4 GPa for the sym-ac_3_–sp^2^ CHC and 551 ± 4 GPa for the sym-ac_3_–sp^2^–sp^3^ CHC (300 K). From a previous study [16], the Young’s modulus of the CHC, which is assumed to be a nanoscale cell solid, can be calculated by the cell wall width via an analytic method. In our cases (wall width is determined as 7 Å), the analytical result is 560 GPa, in a great agreement with our simulation results. 

When the tension starts, both CHCs are stretched quasilinearly under homogeneous and elastic deformation; herein, the initial curve of the sym-ac_3_–sp^2^–sp^3^ CHC deviates a little from that of the sym-ac_3_–sp^2^ CHC. Then, in a certain strain range (from 0.072 to 0.132 for case at 10 K), a yield stage occurs in the deformation of the sym-ac_3_–sp^2^–sp^3^ CHC, which indicates a plastic deformation has initiated. Later in this article, the mechanism therein will be discussed in detail. After the yield step, the sym-ac_3_–sp^2^–sp^3^ curve behaves just like a sym-ac_3_–sp^2^ curve does, even breaking off at the same fracture strength, which is around 61 GPa (56 GPa for case at 300 K), with a retardation measured as around 0.06 in strain for the case at 10 K, equal to the length of the preceding yield stage. Thus, the mechanical behavior of the sym-ac_3_–sp^2^–sp^3^ CHC can be distinguished from that of the sym-ac_3_–sp^2^ CHC by the fracture retardation caused by yield terrace mentioned above. 

**Figure 4:**
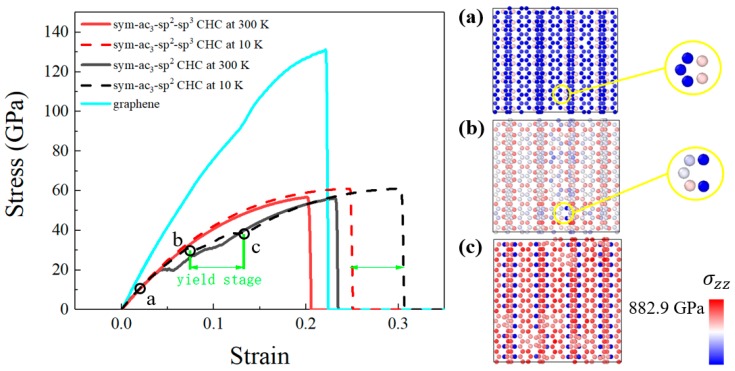
Stress–strain curves of the sym-ac_3_–sp^2^–sp^3^ CHC and sym-ac_3_–sp^2^ CHC. There are three characteristic points representing three different stress states, and corresponding snapshots display the transformation of hinge atoms from sp^3^ to sp^2^, (**a**) for minuscular strain state, (**b**) for the start point of yield terrace, (**c**) for the termination of the yield terrace.

In order to illustrate the mechanism of this yield stage, a slice through some sheets of graphene-like walls has been exhibited in Figure 4. Both cases at 10 K and 300 K exhibit the same yield stage, so in order to avoid random distribution of atoms caused by thermal motion, which will prevent us from observing the detailed transfomation of hinge atoms, we explain the yield process based on the tension at 10 K. The color of atoms characterizes the channel-direction component of the stress on each atom, of which the colormap is linearly related to the value of stress. Through the variation of the channel-direction stress, we can justify whether the hinge atoms interact with other hinge atoms, so that we can determine the bond type of hinge atoms. For hinge atoms with an sp^3^ bond, the neighboring hinge atoms must be bonded with each other, thus exhibiting a high channel-direction stress; while for the case of sp^2^, hinge atoms do not interact with each other, so there will be a low and even zero channel-direction stress on them. In addition, sp^2^ bonding is in-plane (two-dimentional), while sp^3^ is three-dimentional; thus, we can also tell them apart by their geometry and nearest neighbors. Three snapshots at different states of strain, corresponding to three characteristic points in Figure 4, respectively, are displayed. At the beginning of tension (Figure 4a), hinge atoms share most of the distortion energy through the extension of the sp^3^ bond between two hinge atoms, while the sp^3^ configuration remains. However, when the engineering strain reaches around 0.072, some sp^3^ pairs of hinge atoms break up, the distortion energy of graphene-like atoms begins to increase gradually, and simultaneously, the yield stage starts. When the strain attains 0.132, almost all sp^3^ pairs are disconnected, most of the deformation is shared by the atomic cellular walls, and the yield stage terminates at the same time. By contrast, the sym-ac_3_–sp^2^ CHC, without sp^3^ interaction, does not experience such a transformation, and atomic tubes instead of hinge atoms bear most proportions of tensile force. Compared with graphene, although CHC has a lower fracture strength and modulus, it can obtain a higher fracture strain than graphene through this transformation, which may be applied in flexible coating. 

### 4.2. Nanoindentation

At present, nanoindentation is commonly used for the research of mechanical properties of materials on the nanoscale [30,31,32]. There are two main reasons this methodology has been put into widespread usage [33]. As for nanoindentation, the stress applied by indenter is nonhomogeneous and can thus create an elastically physically isolated volume. Secondly, not only the location of elastic instability, but also the slip behaviors can be predicted via nanoindentation. 

**Figure 5:**
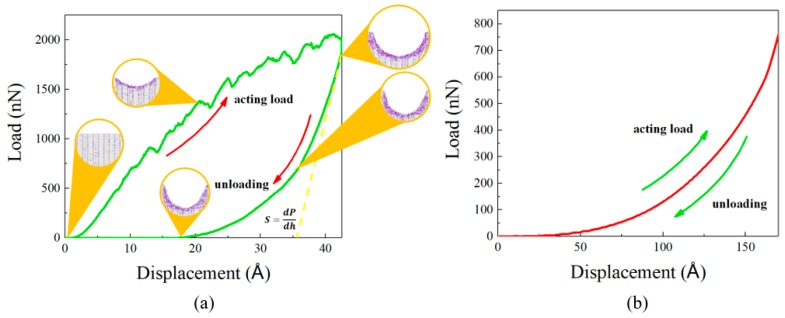
(**a**) The acting–load curve and unloading curve. The slope of tangent line at the initial stage of unloading refers to the contact stiffness $S=\frac{dP}{dh}$. In addition, several snapshots of the morphology of in situ indentation are exhibited here, in which the color of atoms distinguishes different crystal lattices, with grey referring to normal CHC lattice, while purple refers to simple cube lattice, which is a more compact type than the normal one. For comparison between 2D and 3D graphene, the displacement–load curve of graphene under the same simulation condition is displayed in (**b**).

In a previous study [16], nanoindentation behaviors of different sizes of ac_n_–sp^2^ and zz_m_–sp^2^–sp^3^ CHCs were reported. However, the indent depth was small (4 Å) and the unloading process was not displayed. Herein, we simulated the process of nanoindentation in the channel direction of the sym-ac_3_–sp^2^–sp^3^ CHC, an indentation depth of about 40 Å, containing two steps, acting load and unloading, via LAMMPS [28], under the confinement mentioned in the section “Molecular Dynamics Simulations”. The result is displayed in Figure 5, which we used to determine the hardness and Young’s modulus of the CHC in the channel direction. 

Nanoindentation hardness is the average pressure the material can support under an external load, defined as the ratio of indentation load *P* and projected contact area *A_p_* [30]. From the load–displacement curve, the hardness H can be attained using the maximum load as:

(1) $$H=\frac{P_{max}}{A_{p}}.$$

In addition, Young’s modulus can also be calculated indirectly via reduced modulus *E_r_*, which can be expressed as:

(2) $$E_{r}=\frac{\sqrt{\pi }}{2\beta }\frac{S}{\sqrt{A_{p}}},$$

where *S* is termed as contact stiffness, which can be obtained as the slope at the tip of unloading curve, *β* is a constant that depends on the geometry of indenter, for a spherical indenter, *β* = 1. Furthermore, we can determine the substrate’s modulus using following relationship that reflects impacts of both indenter’s and substrate’s moduli:

(3) $$\frac{1}{E_{r}}=\frac{1-\nu_{i}^{2}}{E_{i}}+\frac{1-\nu_{s}^{2}}{E_{s}},$$

where the subscript *i* refers to *indenter*, while *s* refers to *substrate*. 

The nanoindentation hardness is determined as 50.9 GPa in Table 1 and Table 2, which is higher than quartz (9.25 GPa) and even some ion-beam-irradiation hardened metal (over 20 GPa) [34]. In order to analyze the plastic behavior of the ac_3_–sp^2^–sp^3^ CHC in the process of nanoindentation, five snapshots are displayed in Figure 5. In the case of loading, the amounts of atoms which are identified as simple cube lattice are increasing with the dent produced by spherical diamond indenter deepening. This phenomenon indicates a transformation of covalent bonds from a previous normal type to a more compact one. Subsequently, although the indenter retreated and the dent recovering partially in an elastic way, the number of simple cubic atoms is maintained almost the same number as there is when the indenter penetrates the deepest part, which illustrates that a plastic deformation has occurred and cannot rebound. 

## 5. Conclusions

We discussed the elastic and plastic behaviors of sym-ac_3_–sp^2^ CHCs and especially the sym-ac_3_–sp^2^–sp^3^ CHC which is slightly more stable according to our DFT calculation and previous experimental study, using LAMMPS to carry out a typical uniaxial tension and nanoindentation simulation. In the case of uniaxial tension, we discussed the yield stage in the stress–strain curve of the sym-ac_3_–sp^2^–sp^3^ CHC caused by a intriguing transformation of covalent bonds of hinge atoms from strong sp^3^ to comparatively weak sp^2^, and we also determined the Young’s moduli at room temperature, 542 ± 4 GPa for the sym-ac_3_–sp^2^ CHC and 551 ± 4 GPa for the sym-ac_3_–sp^2^–sp^3^ CHC, which are in great agreement with a previous analytical study. Then, in the discussion of nanoindentation, we determined the hardness (50.9 GPa) and Young’s modulus (461 ± 9 GPa) via the load–displacement curve and gave an insight into the plastic deformation caused by the indenter on the nanoscale, which can be explained by the permanent conversion of covalent bonds from sp^3^ type to sp^2^ type. These findings provide an insight into the relationship between the covalent bond type of hinge atoms in carbon honeycomb and the plastic behavior of carbon honeycomb. 

## Figures and Tables

**Table 1:** Hardness and Young’s modulus calculated from nanoindentation. Calculated hardness on the nanoscale.

Projected Contact Area (Å^2^)	Maximum Load (nN/ Å)	Hardness (GPa)
3689	1878.1	50.9

**Table 2:** Hardness and Young’s modulus calculated from nanoindentation. Comparison between Young’s moduli from tension test and nanoindentation.

Contact Stiffness (nN/Å)	Reduced Modulus (GPa)	Young’s Moudulus (From Tension Simulation) (GPa)	Young’s Modulus (From Nanoindentation) (GPa)
228 ± 3	285 ± 4	551 ± 4	461 ± 9

## References

[1] Lee C., Wei X., Kysar J.W., Hone J. (2008). Measurement of the elastic properties and intrinsic strength of monolayer graphene. Science.

[2] Liu F., Ming P., Li J. (2007). Ab initio calculation of ideal strength and phonon instability of graphene under tension. Phys. Rev. B.

[3] Lee G.-H., Cooper R.C., An S.J., Lee S., van der Zande A., Petrone N., Hammerberg A.G., Lee C., Crawford B., Oliver W. (2013). High-strength chemical-vapor–deposited graphene and grain boundaries. Science.

[4] Novoselov K.S., Fal′ko V.I., Colombo L., Gellert P.R., Schwab M.G., Kim K. (2012). A roadmap for graphene. Nature.

[5] Binghui D., Jie H., Hanxing Z., Sheng L., Emily L., Yunfeng S., Qing P. (2017). The normal-auxeticity mechanical phase transition in graphene. 2D Mater..

[6] Young R.J., Kinloch I.A., Gong L., Novoselov K.S. (2012). The mechanics of graphene nanocomposites: A review. Compos. Sci. Technol..

[7] Bae S., Kim H., Lee Y., Xu X., Park J.-S., Zheng Y., Balakrishnan J., Lei T., Ri Kim H., Song Y.I. (2010). Roll-to-roll production of 30-inch graphene films for transparent electrodes. Nat. Nanotechnol..

[8] Nayak T.R., Andersen H., Makam V.S., Khaw C., Bae S., Xu X., Ee P.-L.R., Ahn J.-H., Hong B.H., Pastorin G. (2011). Graphene for controlled and accelerated osteogenic differentiation of human mesenchymal stem cells. ACS Nano.

[9] Nair R.R., Blake P., Blake J.R., Zan R., Anissimova S., Bangert U., Golovanov A.P., Morozov S.V., Geim A.K., Novoselov K.S. (2010). Graphene as a transparent conductive support for studying biological molecules by transmission electron microscopy. Appl. Phys. Lett..

[10] Kuila T., Bose S., Khanra P., Mishra A.K., Kim N.H., Lee J.H. (2011). Recent advances in graphene-based biosensors. Biosens. Bioelectron..

[11] Zhang Y., Huang H. (2008). Stability of single-wall silicon carbide nanotubes – molecular dynamics simulations. Comput. Mater. Sci..

[12] Hong S., Lundstrom T., Ghosh R., Abdi H., Hao J., Jeoung S.K., Su P., Suhr J., Vaziri A., Jalili N. (2016). Highly anisotropic adhesive film made from upside-down, flat, and uniform vertically aligned cnts. ACS Appl. Mater. Interfaces.

[13] Peng Q., Ji W., De S. (2012). Mechanical properties of graphyne monolayers: A first-principles study. Phys. Chem. Chem. Phys..

[14] Krainyukova N.V., Zubarev E.N. (2016). Carbon honeycomb high capacity storage for gaseous and liquid species. Phys. Rev. Lett..

[15] Gao Y., Chen Y., Zhong C., Zhang Z., Xie Y., Zhang S. (2016). Electron and phonon properties and gas storage in carbon honeycombs. Nanoscale.

[16] Zhang Z., Kutana A., Yang Y., Krainyukova N.V., Penev E.S., Yakobson B.I. (2017). Nanomechanics of carbon honeycomb cellular structures. Carbon.

[17] Yang Z., Lan G., Ouyang B., Xu L.-C., Liu R., Liu X., Song J. (2016). The thermoelectric performance of bulk three-dimensional graphene. Mater. Chem. Phys..

[18] Wei Z., Yang F., Bi K., Yang J., Chen Y. (2017). Thermal transport properties of all-sp2 three-dimensional graphene: Anisotropy, size and pressure effects. Carbon.

[19] Pang Z., Gu X., Wei Y., Yang R., Dresselhaus M.S. (2016). Bottom-up design of three-dimensional carbon-honeycomb with superb specific strength and high thermal conductivity. Nano Lett..

[20] Meng F., Chen C., Hu D., Song J. (2017). Deformation behaviors of three-dimensional graphene honeycombs under out-of-plane compression: Atomistic simulations and predictive modeling. J. Mech. Phys. Solids.

[21] Gu X., Pang Z., Wei Y., Yang R. (2017). On the influence of junction structures on the mechanical and thermal properties of carbon honeycombs. Carbon.

[22] Liu Y., Liu J., Yue S., Zhao J., Ouyang B., Jing Y. (2018). Atomistic simulations on the tensile deformation behaviors of three-dimensional graphene. Phys. Status Solidi.

[23] Karfunkel H.R., Dressler T. (1992). New hypothetical carbon allotropes of remarkable stability estimated by mndo solid-state scf computations. J. Am. Chem. Soc..

[24] Park N., Ihm J. (2000). Electronic structure and mechanical stability of the graphitic honeycomb lattice. Phys. Rev. B.

[25] Stuart S.J., Tutein A.B., Harrison J.A. (2000). A reactive potential for hydrocarbons with intermolecular interactions. J. Chem. Phys..

[26] Jia W., Cao Z., Wang L., Fu J., Chi X., Gao W., Wang L.-W. (2013). The analysis of a plane wave pseudopotential density functional theory code on a gpu machine. Comput. Phys. Commun..

[27] Jia W., Fu J., Cao Z., Wang L., Chi X., Gao W., Wang L.-W. (2013). Fast plane wave density functional theory molecular dynamics calculations on multi-gpu machines. J. Comput. Phys..

[28] Plimpton S. (1995). Fast parallel algorithms for short-range molecular dynamics. J. Comput. Phys..

[29] O’Connor T.C., Andzelm J., Robbins M.O. (2015). Airebo-m: A reactive model for hydrocarbons at extreme pressures. J. Chem. Phys..

[30] Li X., Bhushan B. (2002). A review of nanoindentation continuous stiffness measurement technique and its applications. Mater. Charact..

[31] Peng Q., Zhang X., Huang C., Carter E.A., Lu G. (2010). Quantum mechanical study of solid solution effects on dislocation nucleation during nanoindentation. Model. Simul. Mater. Sci. Eng..

[32] Peng Q., Zhang X., Lu G. (2010). Quantum mechanical simulations of nanoindentation of al thin film. Comput. Mater. Sci..

[33] Li J., Van Vliet K.J., Zhu T., Yip S., Suresh S. (2002). Atomistic mechanisms governing elastic limit and incipient plasticity in crystals. Nature.

[34] Takayama Y., Kasada R., Sakamoto Y., Yabuuchi K., Kimura A., Ando M., Hamaguchi D., Tanigawa H. (2013). Nanoindentation hardness and its extrapolation to bulk-equivalent hardness of F82H steels after single- and dual-ion beam irradiation. J. Nucl. Mater..

